# Supplementary material for: Gabapentin Disrupts Binding of Perlecan to the α2δ1 Voltage Sensitive Calcium Channel Subunit and Impairs Skeletal Mechanosensation
Source: Biomolecules. 2022 Dec 12;12(12):1857. doi: 10.3390/biom12121857 (PMC9776037; doi:10.3390/biom12121857)
Supplement: Supplementary file 1 [file biomolecules-12-01857-s001.zip › biomolecules-2055665-supplementary.pdf]

Supplementary information for:

Article

Gabapentin Disrupts Binding of Perlecan to the $\alpha_2\delta_1$ Voltage Sensitive Calcium Channel Subunit and Impairs Skeletal Mechanosensation

Perla C. Reyes Fernandez [1], Christian S. Wright [1], Adrianna N. Masterson [2], Xin Yi [1], Tristen V. Tellman [3], Andrei Bonteanu [3,4], Katie Rust [1], Megan L. Noonan [5], Kenneth E. White [5], Karl J. Lewis [6], Uma Sankar [7], Julia M. Hum [8], Gregory Bix [9], Danielle Wu [3,4], Alexander G. Robling [7], Rajesh Sardar [2], Mary C. Farach-Carson [3,4] and William R. Thompson [1,7,8,*]

[1] Department of Physical Therapy, School of Health and Human Sciences, Indiana University, Indianapolis, IN 46202, USA
[2] Department of Chemistry and Chemical Biology, School of Science, Indiana University, Indianapolis, IN 46202, USA
[3] Department of Diagnostic and Biomedical Sciences, School of Dentistry, The University of Texas Health Science Center at Houston, Houston, TX 77054, USA
[4] Department of Bioengineering, George R. Brown School of Engineering, Rice University, Houston, TX 77005, USA
[5] Department of Medical and Molecular Genetics, School of Medicine, Indiana University, Indianapolis, IN 46202, USA
[6] Meinig School of Biomedical Engineering, Cornell University, Ithaca, NY 14853, USA
[7] Department of Anatomy, Cell Biology and Physiology, School of Medicine, Indiana University, Indianapolis, IN 46202, USA
[8] Division of Biomedical Science, College of Osteopathic Medicine, Marian University, Indianapolis, IN 46222, USA
[9] Departments of Neurosurgery and Neurology, School of Medicine, Tulane University, New Orleans, LA 70112, USA
* Correspondence: thompwil@iu.edu

This file includes:

Supplementary Methods
Supplementary Figures S1 to S8
Supplementary Table S1 to Table S2

Supplementary Methods

S1. Localized surface plasmon resonance experiments

S1.1. Silanization of glass coverslips.

Glass coverslips (25 x 25 mm) were silanized as previously described [1-4]. Briefly, glass coverslips were placed in a glass staining jar, incubated in a 10% RBS 35 alkaline detergent solution at 90 °C and sonicated for 15 min. The coverslips were then rinsed with a copious amount of nanopure water, followed by incubation in a 1:1 (v/v) hydrochloric acid: methanol solution for 30 min at room temperature. After 30 min, the coverslips were rinsed multiple times with nanopure water and then dried overnight in a vacuum oven at 60 °C. The following day, the coverslips were cooled at room temperature and then incubated for 30 min in a 15% (v/v) solution of 3-mercaptopropyltrimethoxysilane (MPTMS) in N_2 purged ethanol. The coverslips were sonicated for 10 min in N_2 purged ethanol three times and dried in a vacuum oven for at least 3 h at 120 °C. The coverslips were stored at 4°C for up to one week.

S1.2. Hill-Langmuir equation ("Specific binding with Hill slope" model).

In the GraphPad Prism software the "Specific binding with Hill slope" model, which is equivalent to the Hill-Langmuir equation, was used to determine the K_D values between α_2 and PLN domains/subdomains (https://www.graphpad.com/guides/prism/latest/curve-fitting/reg_specific_hill.htm). In our saturation binding experiments, we measured specific binding at equilibrium at various concentrations (often 6-12) of the radioligand. The goal is to determine the K_D (ligand concentration that binds to half the receptor sites at equilibrium) and the maximum number of binding sites. In our experiments, the maximum achievable LSPR peak was utilized to determine the K_D values. It is assumed that the LSPR peak ($\Delta\lambda_{LSPR}$) continues to rise with the linear increase in ligand concentration.

The equation follows the model:

$$y = \frac{(\mathrm{Bmax})\mathrm{X}^{\mathrm{h}}}{{K_D}^{\mathrm{h}} + \mathrm{X}^{\mathrm{h}}}$$

Where, **X** = Linear concentration of radioligand (Perlecan domain concentration [M]); **Y** = specific binding ($\Delta\lambda_{LSPR}$ [nm]) obtained for each specific concentration of radioligand**.**

Binding curves were fitted using the least squares regression method, and obtained best-fit values for:

Bmax: Maximum specific binding in the same units as y. It is the specific binding extrapolated to very high concentrations of radioligand, and so its value is almost always higher than any specific binding measured in the experiment.

K_D: Radioligand concentration needed to achieve a half-maximum binding at equilibrium. Expressed in the same units as x.

H: Hill slope coefficient and describes the steepness of the curve. A standard sigmoid dose-response curve has a h= 1.0 and is indicative of non-cooperative (completely independent) binding. When h>1.0, the curve is steeper and indicates positively cooperative binding. Whereas when h<1.0, the curve is more shallow and long (flat) and indicates negatively cooperative binding.

Supplementary Figures

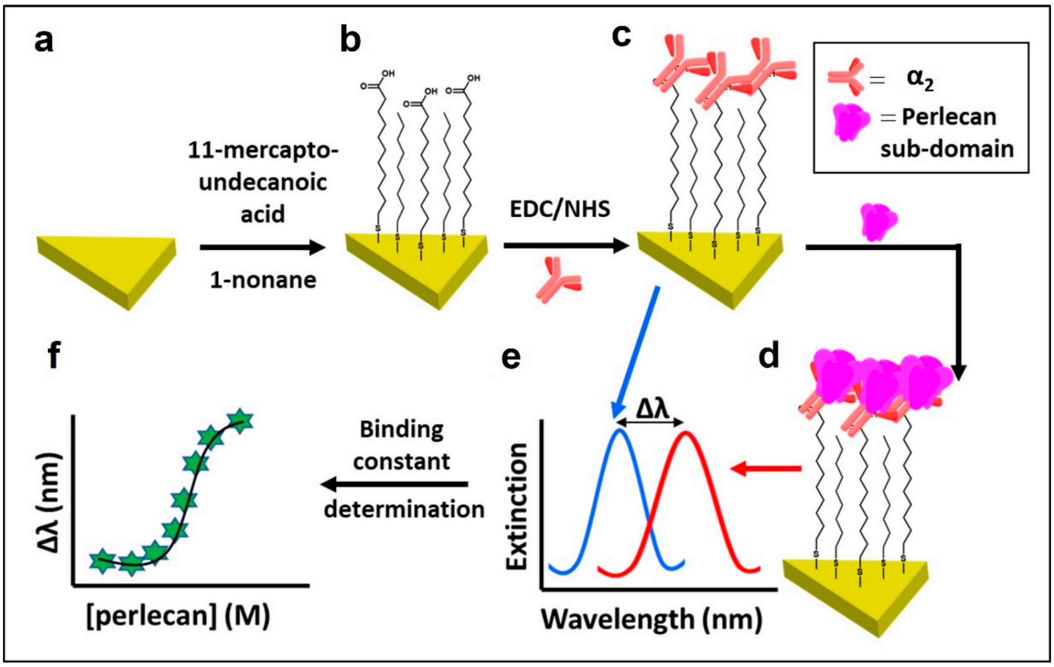

Figure S1. ***Schematic of α_2-functionalized gold nanoprisms for LSPR binding affinity determination of PLN domains***. **(a)** Gold triangular nanoprisms (Au TNPs) were attached onto silanized glass coverslips. **(b)** Nanoprism surface was modified with mercaptoundecanoic acid and nonanethiol solution. **(c)** Au TNPS were incubated in a 1-Ethyl-3-(3-dimethylaminopropyl) carbodiimide/N-hydroxysuccimide (EDC/NHS) solution, and the α_2 portion of the $\alpha_2\delta_1$ subunit attached to the nanoprisms by amide coupling. **(d)** Perlecan (PLN) domains/subdomains were added, and **(e)** Localized surface plasmon resonance (LSPR) wavelengths were measured before (blue curve) and after incubation with PLN using UV-visible spectroscopy (red curve). **(f)** The plot of LSPR peak wavelength shift ($\Delta\lambda$) versus PLN concentration in linear-scale (M) was used to determine binding affinity (K_D) using the Hill-Langmuir equation.

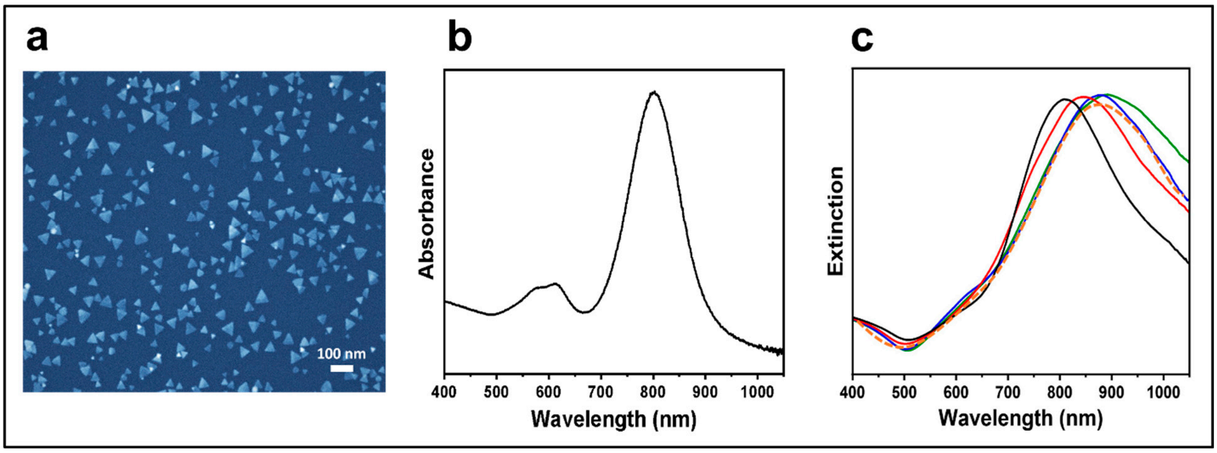

Figure S2. ***LSPR-based sensor characterizations.*** **(a)** Scanning electron microscopy (SEM) image of ~42 nm edge length gold triangular nanoprisms (Au TNPs). **(b)** Representative UV-vis absorption spectrum of ~42 nm edge length Au TNPs in acetonitrile (λ_{LSPR} = 801.9 nm). **(c)** UV-vis extinction spectra show an example of localized surface plasmon resonance (LSPR) shifts used to determine the binding affinity. UV–vis extinction spectra of silanized glass coverslip-bound Au TNPs before surface modification (black curve, 807.9 nm), after functionalization with a self-assembled monolayer (SAM) of 11-mercaptundecanoic acid (1.0 mM): 1-nonanethiol (1.0 µM) (red curve, 846.7 nm), after covalent attachment of 10 ng/mL α_2 (blue curve, 885.7 nm), with PBS (orange dotted curve, 886.8 nm), and finally after adsorption of perlecan domain III (green curve, 899.1 nm).

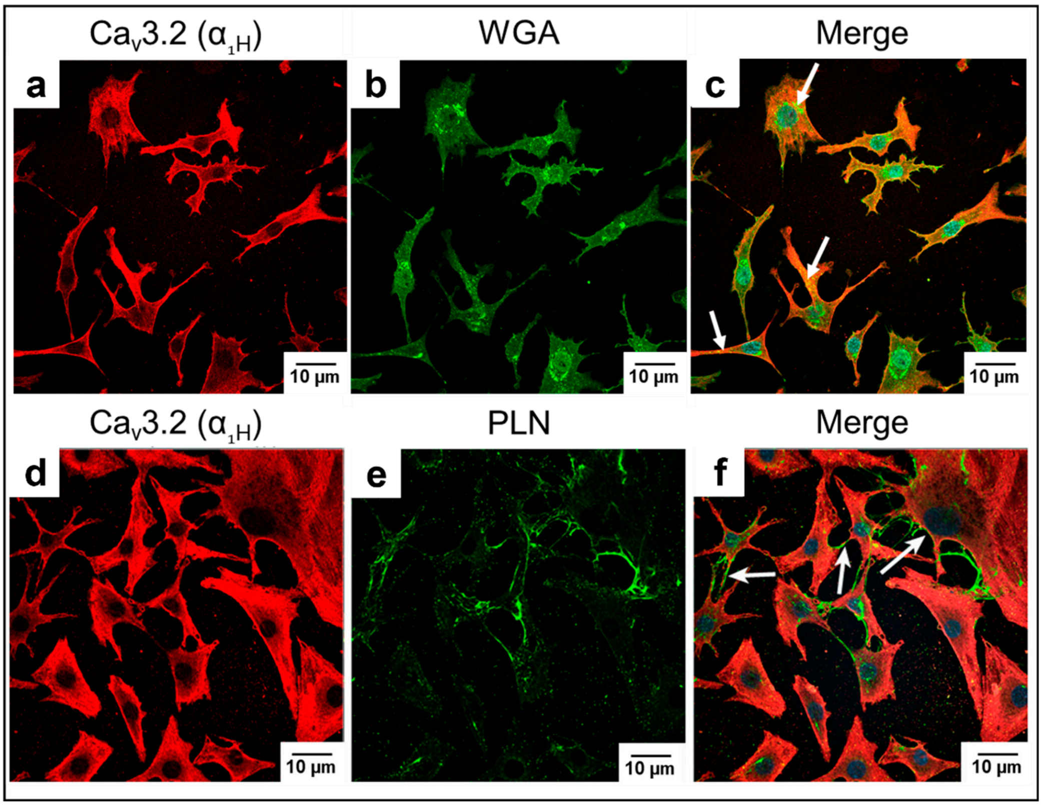

Figure S3. ***Perlecan and Ca$_v$3.2 (α_{1H}) colocalize in osteocyte-like cells.*** Top. MLO-Y4 cells stained for **(a)** Ca$_v$3.2 (α_{1H}) (red) and **(b)** fluorescein conjugated wheat germ agglutinin (WGA) (green). **(c)** The merged image shows overlapping fluorescent signal (white arrows) between α_{1H} and WGA. Bottom, Perlecan (PLN) and α_{1H} colocalize in osteocyte-like cells. MLO-Y4 cells stained for **(d)** Ca$_v$3.2 (α_{1H}) (red) and **(e)** PLN (green). **(f)** The merged image shows overlapping fluorescent signal between α_{1H} and PLN (white arrows).

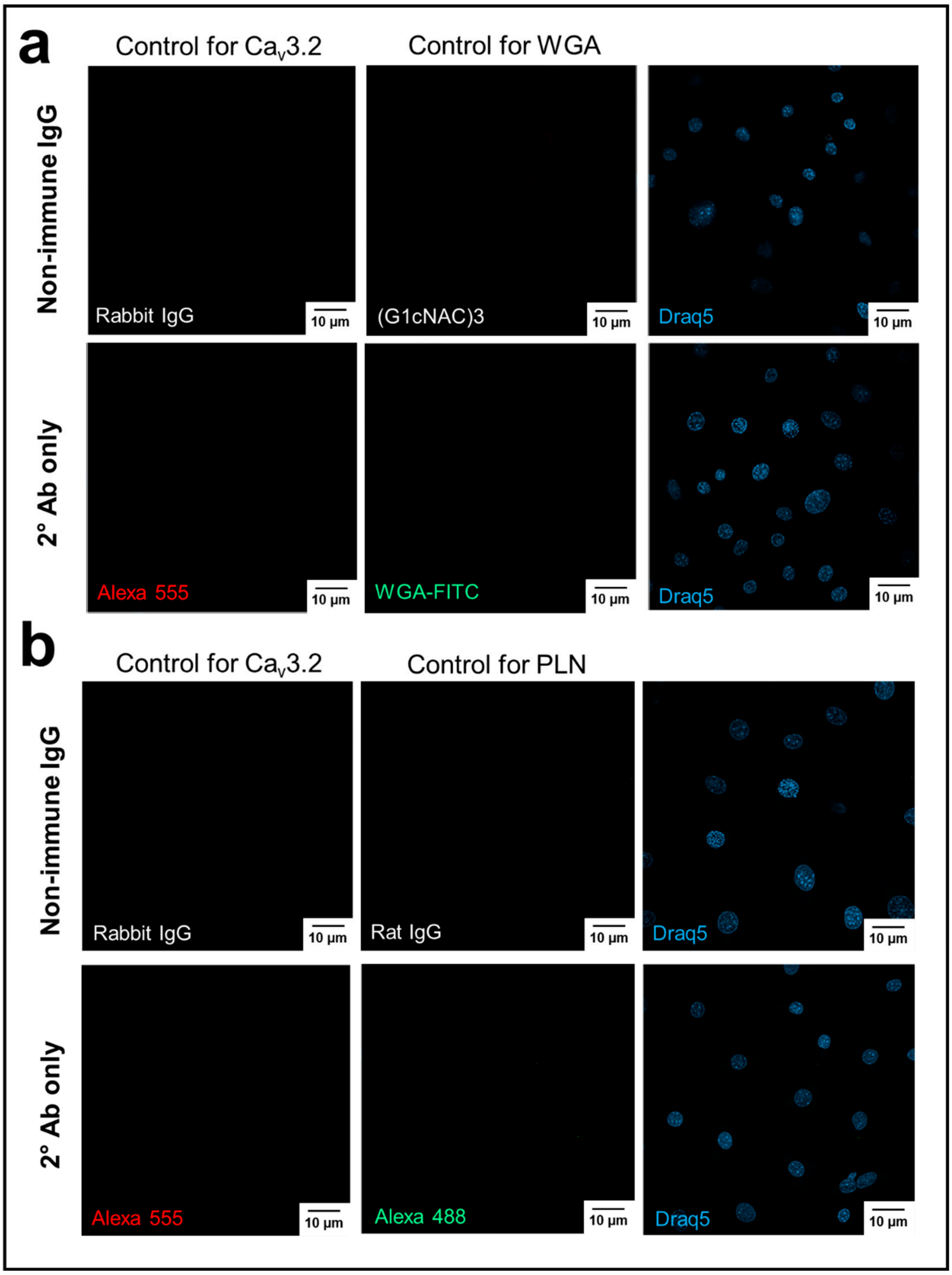

Figure S4. ***Immunofluorescence controls for figure S3.*** MLO-Y4 cells probed for non-immune IgGs or secondary antibodies (2° Ab) only. **(a)** Controls corresponding to Fig S3 a-c; **Top:** Rabbit Normal IgG 1:100 (control for Cav3.2) and Fluorescein conjugated wheat germ agglutinin (WGA-FITC) 1:10 pre-adsorbed with N′N′N′ Triacetylchitotriose [(G1cNAC)3, 20 mM] (control for WGA). **Bottom:** Secondary Antibody Only (control for Ca$_v$3.2) and WGA-FITC 1:10 pre-adsorbed with (G1cNAC)3 (control for WGA). **(b)** Controls corresponding to Fig S3 d-f**; Top:** Rabbit 1:100 (control for Cav3.2) and Rat (control for PLN) Normal IgG. **Bottom:** Secondary Antibody Only. IgG and secondary antibodies were diluted in blocking solution containing 3% BSA, 10% goat serum).

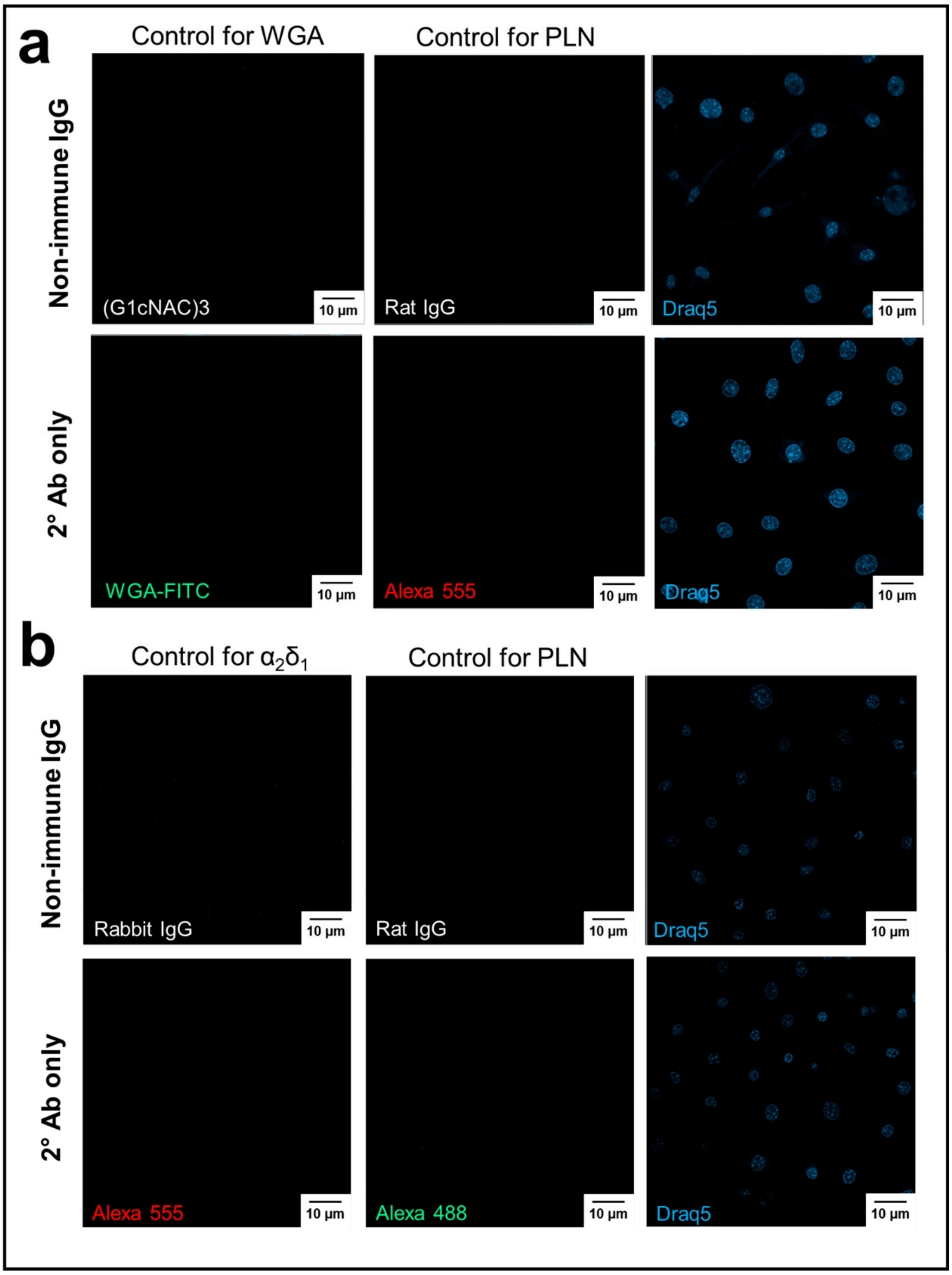

Figure S5. ***Immunofluorescence controls for figure* 2.** MLO-Y4 cells probed for non-immune IgGs or secondary antibodies (2° Ab) only. **(a)** Controls corresponding to Fig 2 a-c; **Top:** Fluorescein conjugated wheat germ agglutinin (WGA-FITC) 1:10 pre-adsorbed with N′N′N′ Triacetylchitotriose [(G1cNAC)3, 20 mM] (control for WGA) and Rat Normal IgG 1:400 (control for PLN), and. **Bottom:** Secondary Antibody Only, WGA-FITC 1:10 pre-adsorbed with (G1cNAC)3 and Alexa 555 (PLN). **(b)** Controls corresponding to Fig 2 d-f**; Top:** Rabbit 1:50 (control for $\alpha_2\delta_1$) and Rat (control for PLN) Normal IgG. **Bottom:** Secondary Antibody Only. IgG and secondary antibodies were diluted in blocking solution containing 3% BSA and 10% goat serum.

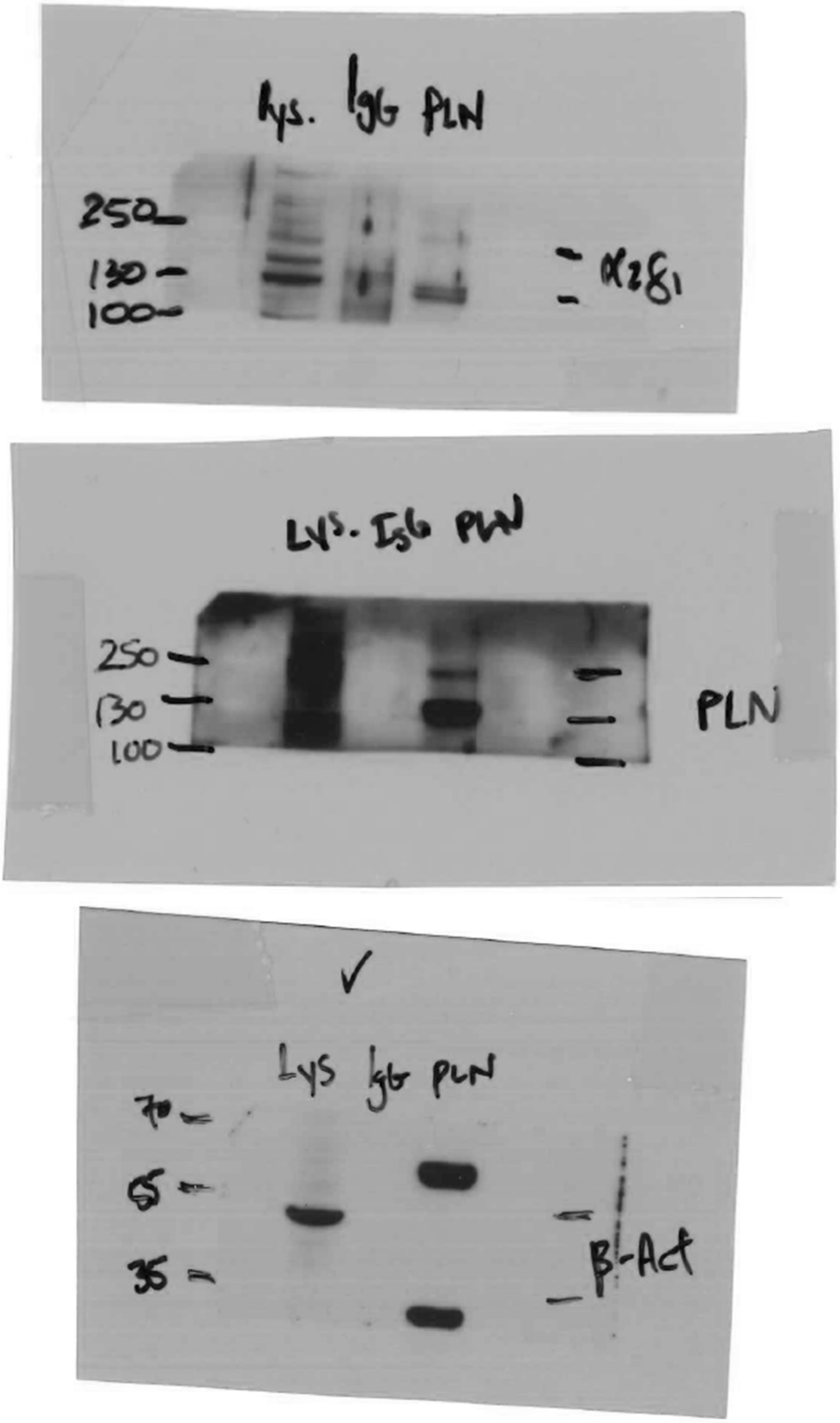

Figure S6. ***Original, uncropped, unadjusted Western images from Co-IP experiment shown in figure 2g.*** Lys; MLO-Y4 lysates; IgG: Immunoglobulin G and PLN: Perlecan. **Top:** Blot probed with an antibody to detect $\alpha_2\delta_1$. **Middle:** Blot probed with an antibody to detect Perlecan. **Bottom:** Blot probed with an antibody to detect β-actin (loading control).

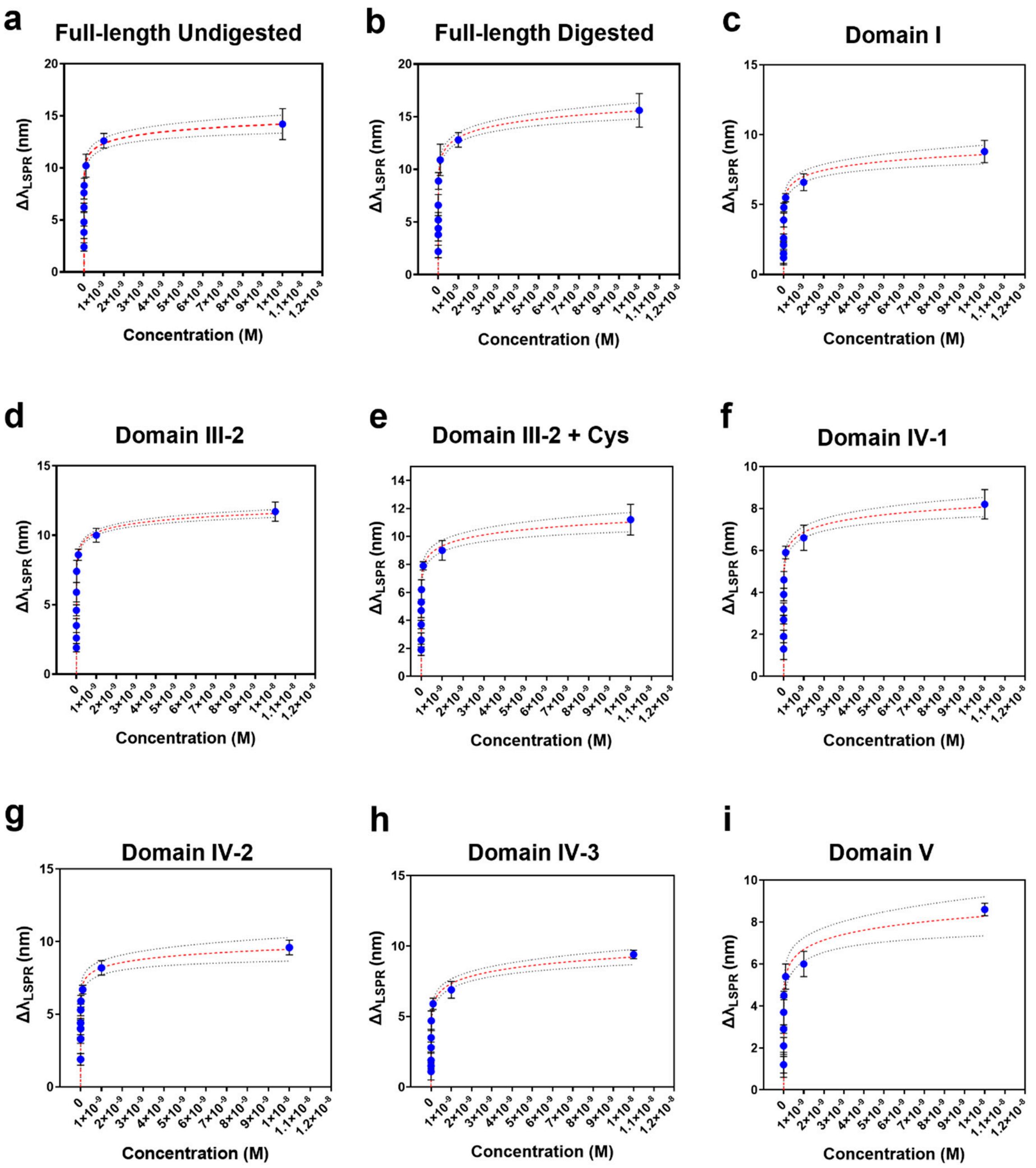

Figure S7. ***Dissociation constant curves for the*** α_2 ***portion of the*** $\alpha_2\delta_1$ ***subunit and PLN***. α_2-functionalized sensors were incubated with a solution containing different concentrations (1×10^{-16} to 1×10^{-8} M) of full-length perlecan (PLN) or each of PLN domains (Dm). Binding curves were constructed for **(a)** Full-length PLN (undigested) and **(b)** enzymatically digested with heparinase and chondroitinase, **(c)** PLN Dm I **(d)** Dm III-2, **(e)** Dm III-2 with cysteine, **(f-h)** PLN Dm IV-1, -2, and -3, and **(i)** PLN Dm V. Each data point (blue) represents the average shift in the LSPR dipole peak position ($\Delta\lambda_{LSPR}$) values of six measurements (Mean± SD). Fitting curves (red dashed line) were developed by plotting $\Delta\lambda_{LSPR}$ versus PLN [or PLN subdomains] concentration in mol/L (M). Confidence bands (95%) showing the likely location of a true curve are depicted with black lines. Data was analyzed using the Hill-Langmuir equation (specific binding Hill equation model in Graph Pad) to determine K_D values [5-7]. The raw data corresponding to these graphs can be found in **Table S1.** Hill coefficient and maximum specific binding values are shown in **Table S2**.

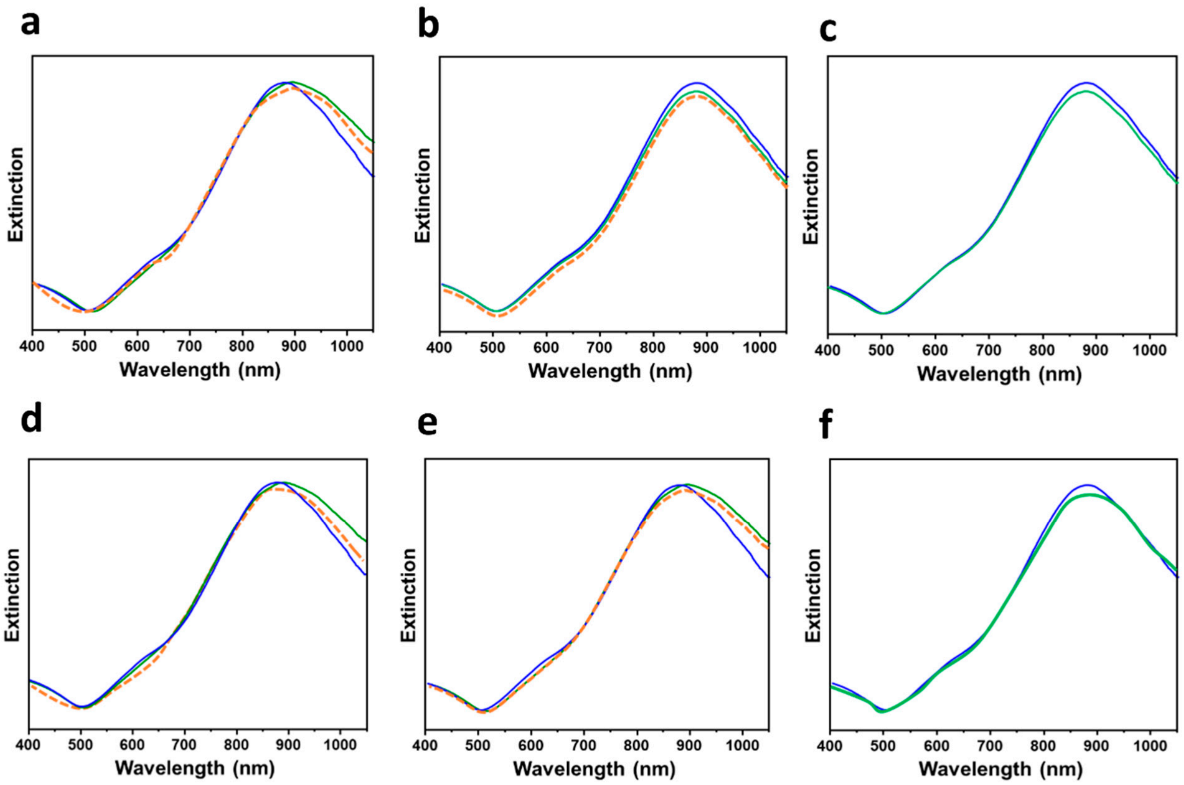

Figure S8. ***UV-vis extinction spectra showing examples of localized surface plasmon resonance (LSPR) based interactions among α_2-functionalized sensors, perlecan, and gabapentin.*** All experiments start with α_2-sensors (blue curve, 885.7 nm). Perlecan (PLN), Gabapentin (GBP) or a mixture of both are added to the nanoprisms and shifts in the LSPR dipole peak position ($\Delta\lambda_{LSPR}$) are shown in the graphs. ***(a)*** Experiment 1, α_2-sensors were functionalized with full length PLN (green curve, 900.1 nm, $\Delta\lambda_{LSPR}$= +14.4 nm) followed by GBP (orange dotted curve, 896.0 nm, $\Delta\lambda_{LSPR}$= -4.1 nm). ***(b)*** Experiment 2, α_2 sensors were functionalized with GBP (green curve, 891.5 nm, $\Delta\lambda_{LSPR}$= +5.8 nm) followed by Full length PLN (orange dotted curve, 891.6 nm, $\Delta\lambda_{LSPR}$= +0.1 nm). ***(c)*** Experiment 3, α_2 were functionalized with full length PLN + GBP (green curve, 888.4 nm, $\Delta\lambda_{LSPR}$ = +2.7nm). Similar experiments were conducted with PLN Dm III-2 and GBP, ***(d)*** Experiment 4, α_2 sensors (blue curve, 885.7 nm) were functionalized with PLN Dm III-2 (green curve, 898.4 nm, $\Delta\lambda_{LSPR}$ =+12.7 nm), followed by GBP (orange dotted curve, 894.1 nm, $\Delta\lambda_{LSPR}$ = -4.3nm). ***(e)*** Experiment 5, α_2 sensors were functionalized with GBP (green curve, 891.1 nm, $\Delta\lambda_{LSPR}$ =+5.4nm) followed by PLN Dm III-2 (orange dotted curve, 891.5 nm, $\Delta\lambda_{LSPR}$ = +0.4nm). ***(f)*** Experiment 6, α_2-sensors were functionalized with PLN Dm III-2 + GBP (green curve, 890.6 nm, $\Delta\lambda_{LSPR}$ =+4.9nm).

Supplementary Tables

Table S1. *$\Delta\lambda_{LSPR}$ in α_2-sensors (nm) for each of concentration of PLN domains/sub-domains tested*

A. PLN full length undigested

Concentration (M)	Full Length Undigested $\Delta\lambda_{LSPR}$ (nm)	Standard Deviation (nm)
$1x10^{-8}$	14.2	1.5
$1x10^{-9}$	12.6	0.7
$1x10^{-10}$	10.2	1.1
$1x10^{-11}$	8.3	0.7
$1x10^{-12}$	7.6	0.6
$1x10^{-13}$	6.2	0.4
$1x10^{-14}$	4.8	0.9
$1x10^{-15}$	3.8	0.6
$1x10^{-16}$	2.4	0.4

B. PLN full length digested

Concentration (M)	Full Length Digested $\Delta\lambda_{LSPR}$ (nm)	Standard Deviation (nm)
$1x10^{-8}$	15.6	1.6
$1x10^{-9}$	12.8	0.7
$1x10^{-10}$	10.9	1.5
$1x10^{-11}$	8.9	0.8
$1x10^{-12}$	6.6	1
$1x10^{-13}$	5.2	0.7
$1x10^{-14}$	4.4	0.7
$1x10^{-15}$	3.8	0.6
$1x10^{-16}$	2.2	0.6

C. PLN Dm I

Concentration (M)	Domain-I $\Delta\lambda_{LSPR}$ (nm)	Standard Deviation (nm)
$1x10^{-8}$	8.8	0.8
$1x10^{-9}$	6.6	0.6
$1x10^{-10}$	5.5	0.3
$1x10^{-11}$	4.8	0.3
$1x10^{-12}$	3.9	0.5
$1x10^{-13}$	2.6	0.3
$1x10^{-14}$	2.1	0.3
$1x10^{-15}$	1.5	0.7
$1x10^{-16}$	1.2	0.5

D. PLN Dm III-2

Concentration (M)	Domain-III-2 $\Delta\lambda_{LSPR}$ (nm)	Standard Deviation (nm)
$1x10^{-8}$	11.7	0.7
$1x10^{-9}$	10	0.5
$1x10^{-10}$	8.6	0.4
$1x10^{-11}$	7.4	0.8
$1x10^{-12}$	5.9	0.7
$1x10^{-13}$	4.6	0.4
$1x10^{-14}$	3.5	0.5
$1x10^{-15}$	2.6	0.4
$1x10^{-16}$	1.9	0.3

E. PLN Dm III-2 + Cysteine

Concentration (M)	Domain III-2 + cys $\Delta\lambda_{LSPR}$ (nm)	Standard Deviation (nm)
$1x10^{-8}$	11.2	1.1
$1x10^{-9}$	9	0.7
$1x10^{-10}$	7.9	0.3
$1x10^{-11}$	6.2	0.7
$1x10^{-12}$	5.3	0.2
$1x10^{-13}$	4.7	0.5
$1x10^{-14}$	3.7	0.3
$1x10^{-15}$	2.6	0.5
$1x10^{-16}$	1.9	0.4

F. PLN Dm IV-1

Concentration (M)	Domain IV-1 $\Delta\lambda_{LSPR}$ (nm)	Standard Deviation (nm)
$1x10^{-8}$	8.2	0.7
$1x10^{-9}$	6.6	0.6
$1x10^{-10}$	5.9	0.3
$1x10^{-11}$	4.6	0.4
$1x10^{-12}$	3.9	0.3
$1x10^{-13}$	3.2	0.3
$1x10^{-14}$	2.7	0.2
$1x10^{-15}$	1.9	0.3
$1x10^{-16}$	1.3	0.5

G. PLN Dm IV-2

Concentration (M)	Domain IV-2 $\Delta\lambda_{LSPR}$ (nm)	Standard Deviation (nm)
$1x10^{-8}$	9.6	0.5
$1x10^{-9}$	8.2	0.5
$1x10^{-10}$	6.7	0.3
$1x10^{-11}$	5.9	0.4
$1x10^{-12}$	5.3	0.4
$1x10^{-13}$	4.4	0.3
$1x10^{-14}$	4	0.6
$1x10^{-15}$	3.3	0.3
$1x10^{-16}$	1.9	0.4

H. PLN Dm IV-3

Concentration (M)	Domain IV-3 $\Delta\lambda_{LSPR}$ (nm)	Standard Deviation (nm)
$1x10^{-8}$	9.4	0.3
$1x10^{-9}$	6.9	0.6
$1x10^{-10}$	5.9	0.4
$1x10^{-11}$	4.7	0.7
$1x10^{-12}$	3.5	0.6
$1x10^{-13}$	2.8	0.4
$1x10^{-14}$	1.9	0.6
$1x10^{-15}$	1.5	0.4
$1x10^{-16}$	1.1	0.6

I. PLN Dm V

Concentration (M)	Domain-V $\Delta\lambda_{LSPR}$ (nm)	Standard Deviation (nm)
$1x10^{-8}$	8.6	0.3
$1x10^{-9}$	6	0.6
$1x10^{-10}$	5.4	0.6
$1x10^{-11}$	4.5	0.2
$1x10^{-12}$	3.7	0.7
$1x10^{-13}$	2.9	0.2
$1x10^{-14}$	2.1	0.4
$1x10^{-15}$	1.2	0.4
$1x10^{-16}$	1.2	0.6

$\Delta\lambda_{LSPR}$ = Shift in Localized Surface Plasmon Resonance (LSPR) peak (nm)

Table S2. *Hill coefficients and Bmax values obtained from affinity experiments between $\alpha_2\delta_1$ and Perlecan*

Perlecan Domain / Subdomain	Hill coefficient (h)	Bmax (nm)
Undigested Full Length	0.121	26.7
Digested Full Length	0.122	38.67
Domain I	0.122	27.84
**Domain III-2**	**0.151**	**17.15**
Domain III-2 (w/cystine)	0.105	33.19
Domain IV-I	0.116	19
Domain IV-2	0.086	33.1
Domain IV-3	0.124	41.89
Domain V	0.111	43.84

Bmax = Maximum specific binding extrapolated to very high concentrations of radioligand

References

1. Liyanage, T.; Sangha, A.; Sardar, R., Achieving biosensing at attomolar concentrations of cardiac troponin T in human biofluids by developing a label-free nanoplasmonic analytical assay. *Analyst (Cambridge, United Kingdom)* **2017,** 142, (13), 2442-2450.
2. Liyanage, T.; Masterson, A. N.; Oyem, H. H.; Kaimakliotis, H.; Nguyen, H.; Sardar, R., Plasmoelectronic-Based Ultrasensitive Assay of Tumor Suppressor microRNAs Directly in Patient Plasma: Design of Highly Specific Early Cancer Diagnostic Technology. *Analytical Chemistry (Washington, DC, United States)* **2019,** 91, (3), 1894-1903.
3. Masterson, A. N.; Liyanage, T.; Berman, C.; Kaimakliotis, H.; Johnson, M.; Sardar, R., A novel liquid biopsy-based approach for highly specific cancer diagnostics: mitigating false responses in assaying patient plasma-derived circulating microRNAs through combined SERS and plasmon-enhanced fluorescence analyses. *Analyst (Cambridge, United Kingdom)* **2020,** 145, (12), 4173-4180.
4. Hati, S.; Langlais, S. R.; Masterson, A. N.; Liyanage, T.; Muhoberac, B. B.; Kaimakliotis, H.; Johnson, M.; Sardar, R., Photoswitchable machine-engineered plasmonic nanosystem with high optical response for ultrasensitive detection of microRNAs and proteins adaptively. *Analytical Chemistry (Washington, DC, United States)* **2021,** 93, (41), 13935-13944.
5. Wienken, C. J.; Baaske, P.; Rothbauer, U.; Braun, D.; Duhr, S., Protein-binding assays in biological liquids using microscale thermophoresis. *Nature Communications* **2010,** 1, (1), 1-7.
6. Hulme, E. C.; Trevethick, M. A., Ligand binding assays at equilibrium: validation and interpretation. *British Journal of Pharmacology* **2010,** 161, (6), 1219-1237.
7. Masterson, A. N.; Muhoberac, B. B.; Gopinadhan, A.; Wilde, D. J.; Deiss, F. T.; John, C. C.; Sardar, R., Multiplexed and high-throughput label-free detection of RNA/spike protein/IgG/IgM biomarkers of SARS-CoV-2 infection utilizing nanoplasmonic biosensors. *Analytical Chemistry (Washington, DC, United States)* **2021,** 93, (25), 8754-8763.
